# MicroRNA-33a-3p suppresses cell migration and invasion by directly targeting PBX3 in human hepatocellular carcinoma

**DOI:** 10.18632/oncotarget.9886

**Published:** 2016-06-07

**Authors:** Shu-Yan Han, Hai-Bo Han, Xiu-Yun Tian, Hong Sun, Dong Xue, Can Zhao, Shan-Tong Jiang, Xi-Ran He, Wen-Xian Zheng, Jing Wang, Li-Na Pang, Xiao-Hong Li, Ping-Ping Li

**Affiliations:** ^1^ Key Laboratory of Carcinogenesis and Translational Research (Ministry of Education), Peking University Cancer Hospital & Institute, Beijing 100142, PR China; ^2^ Department of Integrative Medicine and Geriatric Oncology, Peking University Cancer Hospital & Institute, Beijing 100142, PR China; ^3^ Department of Biobank, Peking University Cancer Hospital & Institute, Beijing 100142, PR China; ^4^ Department of Hepato-Pancreato-Biliary Surgery, Peking University Cancer Hospital & Institute, Beijing 100142, PR China

**Keywords:** hepatocellular cancer (HCC), migration, metastasis, miR-33a-3p, PBX3

## Abstract

MicroRNAs (miRNAs) have been shown to function as either oncogenes or tumor suppressors by negatively regulating target genes involved in tumor initiation and progression. In this study, we demonstrated that down-regulation of miR-33a-3p in human primary hepatocellular cancer (HCC) specimens was significantly associated with metastases and poor survival. Over-expression of miR-33a-3p in HepG2 cells remarkably suppressed not only cell growth, migration and invasion, but also tumor growth and metastases in the chick embryo chorioallantoic membrane (CAM) assay, and down-regulated Pre-B-Cell Leukemia Homeobox 3 (*PBX3*) expression. Conversely, inhibition of miR-33a-3p in Bel-7402 cells resulted in increased of cell growth, spreading and invasion. Furthermore, rescue experiments by over-expression *PBX3* completely eliminated the inhibitory effects of miR-33a-3p on tumor growth and metastasis, both *in vitro* and *in vivo*. The luciferase assay showed that 3′-untranslated regions (3′-UTRs) of *PBX3* were inhibited significantly by miR-33a-3p, while mutations in the miR-33a-3p pairing residues rescued the luciferase expression. Taken together, our findings suggest that miR-33a-3p suppressed the malignant phenotype while also inhibiting *PBX3* expression in hepatocellular cancer, implying that miR-33a-3p may be a promising biomarkers and therapy target for HCC intervention.

## INTRODUCTION

Hepatocellular cancer (HCC) is characterized by significant morbidity and high mortality rates worldwide. According to the World Health Organization (WHO; GLOBOCAN 2012), HCC is the second leading cause of cancer-related mortality [1], and the incidence rates are expected to increase in coming years. HCC has a poor prognosis as a result of a low detection rate at the curable stages and a high rate of recurrence.

MicroRNAs (miRNAs) are small noncoding single-stranded RNAs consisting of 21–25 nucleotides that post-transcriptionally regulate gene expression [2, 3]. Mature miRNAs can bind to 3′-untranslated region (UTR), 5′-UTR, or coding regions of messenger RNA (mRNA), and in turn trigger mRNA degradation or inhibition of translation [3, 4]. Thousands of miRNAs have been identified in the human genome [5], and many target genes of cancer-related miRNAs have been identified as associated with tumorigenesis, tumor growth, angiogenesis and metastasis [6].

Increasing experimental evidence backs up the idea of aberrant miRNA expression in cancer pathogenesis. Since Murak et al. [7] first reported that HCC exhibited an abnormal expression pattern of miRNAs, several studies have demonstrated that miRNAs play an important regulatory role in hepatocarcinogenesis and malignant transformation [8, 9].

Members of miR-33 family are intronic miRNAs that are located within the sterol regulatory element-binding protein (SREBP) genes and function as regulators of glucose and lipid metabolism [10, 11]. The functional relevance of miR-33a in cancer was established in 2012, and it was shown to act as a tumor suppressor in lymphoma and colon carcinoma [12]. MiR-33a is also regarded as a good prognostic indicator of overall survival in pancreatic cancer patients [13] and as a suppressor of bone metastasis in lung cancer [14]. While the functional role of miR-33a has been highly investigated, the effect of its passenger strand, miR-33a-3p, has not been well addressed to date.

MiR-33a-3p (MIMAT0004506), whose previous name is miR-33a*, shares a pre-miRNA hairpin with miR-33a (MIMAT0000091). MiR-33a-3p was reported to have a higher expression level than miR-33a in liver tissue [10]. However, there is no data available about the functional relevance and expression profile of miR-33a-3p in HCC or other cancers. In the present study, we detected the expression profile of miR-33a-3p in HCC, investigated its role in tumor growth and metastasis, and characterized its target gene.

## RESULTS

### Down-regulation of miR-33a-3p is associated with the metastatic properties of human HCC cells

**Figure 1:**
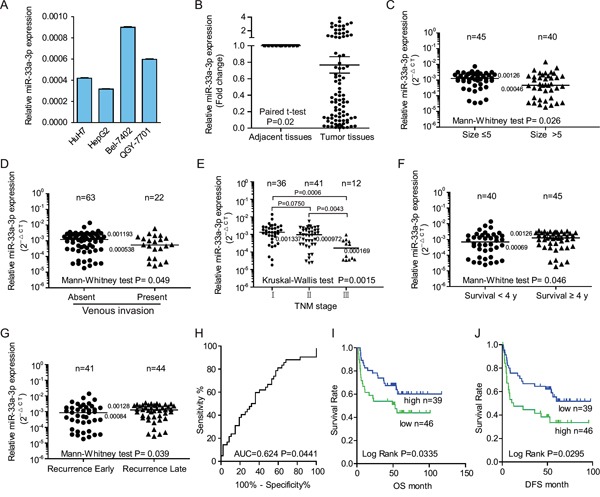
The down-regulation of miR-33a-3p was associated with metastases in hepatocellular carcinoma (HCC) cell lines and tumor tissues from hepatocellular carcinoma patients **A.** Comparison of miR-33a-3p expression levels in HCC cell lines. The expression levels of miR-33a-3p were determined by qRT-PCR and normalized to *U6*. **B.** Expression levels of miR-33a-3p in HCC and matched adjacent nontumorous tissues. **C–G.** The expressions of miR-33a-3p were compared between tumor size (C), venous invasion (D), TNM stage (E), survival years (F), and recurrence (G) in HCC tissues. **H.** The ROC analysis for z-scores of miR-33a-3p expression to evaluate the survival status. **I** and **J.** Overall survival analysis (I) and the tumor-free survival (J) of patients were compared based on the expression levels of miR-33a-3p in HCC tumor tissues. Horizon lines in B to G indicate the median values of each group.

To investigate the functional relevance of miR-33a-3p in HCC, we first examined the expression level of miR-33a-3p in HCC cells with different metastatic properties by real-time polymerase chain reaction (PCR). As indicated in Figure 1A, miR-33a-3p expression is relatively lower in cells with high metastatic potential (HepG2 and HuH7 cells) than those with low metastatic ability (Bel-7402 and QGY7701). In line with our results, other reports also demonstrated that HepG2 and HuH7 cells are highly metastatic HCC cell lines [15, 16], while Bel-7402 and QGY7701 cells are with a less metastatic potential [16]. These data suggest that down-regulation of miR-33a-3p correlates with the high metastatic phenotype in HCC cells.

### Decreased expression of miR-33a-3p is associated with higher invasion properties, low 4-year survival rate, and early recurrence

To determine whether miR-33a-3p was associated with metastasis in clinical samples, we detected miR-33a-3p expression in 89 paired HCC and normal tissues. Not surprisingly, the expression levels of miR-33a-3p were significantly lower in tumor tissues than in the paired adjacent tissues (*P* = 0.02; Figure 1B). We then analyzed the relationship between miR-33a-3p expression and clinicopathologic characteristics in a total of 85 cases with long-term follow-up of patients. These data are summarized in Table 1 and Figure 1C–1F. As shown in Figures 1C–1G, low miR-33a-3p expression levels were significantly associated with big tumor size (*P* = 0.026), venous invasion (n = 22; median value 0.0005 vs. 0.0012; *P* = 0.049; Figure 1D), advanced TNM stage (Figure 1E), overall 4-year survival rate (*P* = 0.046, Figure 1F) and early recurrence in 85 HCC patients (*P* = 0.039, Figure 1G). The above data demonstrate that the low expression levels of miR-33a-3p in HCC tissues are correlated with higher invasion properties, low 4-year survival rate and early recurrence. However, there was no significant correlation between miR-33a-3p expression and gender, age at surgery, or hepatic cirrhosis (Table 1).

**Table 1:** Relationships between the expression of miR-33a-3p mRNA and the clinicopathologic features in 85 HCC patients

Variable		Case no.	miR-33a-3p expression (RQ: 2^−ΔCt^)		*P-*value ^a^
			Median	Range	
*Gender*	Male	74	0.001138	0.000018-0.01393-	0.091
	Female	11	0.0001755	0.000031-0.003562	
*Age* (year)	≤60	63	0.000972	0.000024-0.01393	0.537
	>60	22	0.001141	0.000018-0.007455	
*Cirrhosis*	Absent	27	0.001385	0.000031-0.003277	0.082
	Present	58	0.0008595	0.000018-0.01393	
*Size* (cm)	≤5	45	0.00126	0.000035-0.007455	0.026
	>5	40	0.000465	0.000018-0.01393	
*Venous invasion*	Absent	63	0.001193	0.000018-0.01393	0.049
	Present	22	0.000538	0.000024-0.005797	
*TNM stage*	I	36	0.001337	0.000018-0.01393	0.0015
	II	41	0.000972	0.000024-0.005797	
	III	12	0.000169	0.000033-0.000926	
*Survival* (year)	<4	40	0.0006955	0.000024-0.01393	0.046
	≥4	45	0.00126	0.000018-0.003562	
*Recurrence*	Early ^b^	41	0.000847	0.000018 - 0.01393	0.039
	Late ^c^	44	0.001281	0.000026 - 0.003562	

a Mann–Whitney test for two groups and Kruskal-Wallis test for 3 or more independent samples.

b Recurrences occur within 2 years of diagnosis.

c No recurrences within 4 years of diagnosis.

### Decreased level of miR-33a-3p expression predicts a short survival time

To choose a standard z-score to define over-expression in HCC, we calculated standardized scores (z-scores) for the expression levels of miR-33a-3p. Receiver-operator curve (ROC) analysis for z-scores was performed to evaluate the survival status (area under the curve [AUC] = 0.624, *P* = 0.0441, Figure 1H). The z-score value of −0.2698 was selected after estimating the optimal Youden index-based cut-off point and Kaplan−Meier survival curves for HCC patients (shown in Figures 1I and 1J). The patients with low miR-33a-3p expression displayed both shorter overall survival periods (*P* = 0.0335, Figure 1I) and tumor-free survival (*P* = 0.0295, Figure 1J), suggesting that low levels of miR-33a-3p expression are associated with poor survival of HCC. However, multivariate Cox proportional hazard model analysis failed to indicate that the expression of miR-33a-3p is an independent prognostic factor (Supplementary Table S1).

### Over-expression of miR-33a-3p inhibits cell growth, spreading, migration and invasion in HepG2

**Figure 2:**
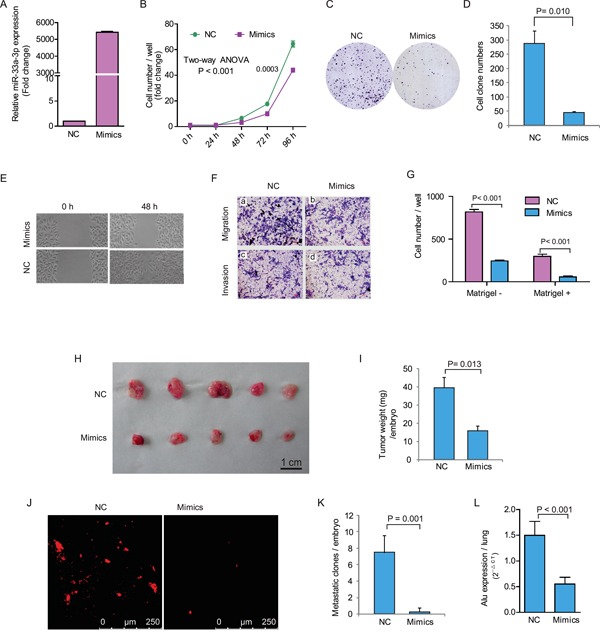
Ectopic expression of miR-33a-3p inhibited HepG2 cell proliferation, motility, migration and invasion **A.** The expression of miR-33a-3p after transfection of mimics into HepG2 cells. **B–D.** The effects of over-expression of miR-33a-3p on HepG2 cell proliferation (B) and colony formation (C, D). **E.** The cell motility was measured using a wound-healing assay. **F.** The cell migration and invasion were measured using the Transwell chamber assay without (a, b) or with Matrigel (c, d). **G.** Quantitative results are illustrated for F. **H–I.** *In vivo* tumor growth was evaluated by chick embryo chorioallantoic membrane (CAM) assay (H). Histogram displays of tumor weights (I). **J–K.** Tumor metastasis was identified as Dil-positive cell clones in chick embryo lung under a fluorescence microscope (J), and the quantitative results are illustrated in (K). **L.** Intravasation of HepG2 cells into chicken embryo lung tissues was determined by human-specific *Alu* sequence expression. Data of *in vivo* represent the mean ± SD of five chick embryos.

To further understand the function of miR-33a-3p in HCC cells, we transfected the highly metastatic cells (HepG2) with miR-33a-3p mimics and then measured the changes in cell proliferation, spreading, migration and invasion abilities *in vitro*. As shown in Figure 2A, the expression level of miR-33a-3p increased significantly after the mimics' transfection at 24 h. Following with the increased miR-33a-3p expression, HepG2 cell proliferation (Figure 2B) and colony formation were both remarkably inhibited (Figure 2C and 2D). The wound-healing assay indicated that the spreading of miR-33a-3p over-expressing HepG2 cells was much slower than in the control cells (Figure 2E). Compared with control cells, the migration and invasion abilities of HepG2 cells transfected with miR-33a-3p mimics were dramatically decreased (*P* < 0.001) as revealed by Boyden chamber assays (Figure 2F and 2G). The above results demonstrate that the ectopic expression of miR-33a-3p suppresses cell proliferation, spreading, migration and invasion *in vitro*, indicating that miR-33a-3p may play a key role in inhibiting cancer growth and metastasis of HCC cells.

Next, we adopted a modified chick embryo chorioallantoic membrane (CAM) assay to assess the influence of miR-33a-3p over-expression on growth and metastatic properties of HepG2 cells *in vivo*. As shown in Figure 2H and 2I, the tumor growth of HepG2 cells on CAM was significantly decreased by 60% after the transfection of miR-33a-3p mimics (miR-33a-3p 15.8 ± 2.9 mg vs. control 39.5 ± 6.0 mg, *P* = 0.013). Furthermore, there were no metastatic clones formed except for few scattered cells in the chick embryo lungs were detected upon miR-33a-3p over-expression. On the contrary, the number of metastatic clones (> 20 cells) of the control group was 7.5 ± 1.0 per embryo (Figure 2J and 2K). Quantitative determination of human *Alu* expression in CAM lungs by quantitative reverse transcription PCR (qRT-PCR) showed that the intravasation of HepG2 cells was prominently abrogated by miR-33a-3p mimics (Figure 2L). These data reveal that miR-33a-3p suppressed both tumor growth and metastasis of HCC cells.

### Inhibition of miR-33a-3p enhanced cell growth, motility and invasion in Bel-7402 cells

**Figure 3:**
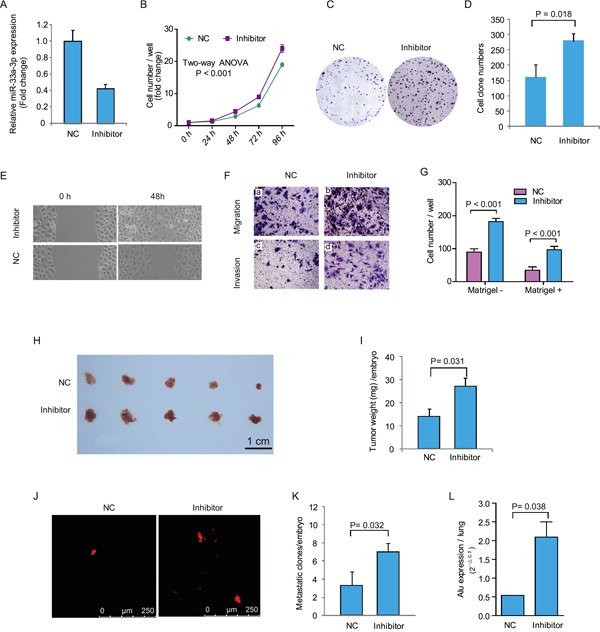
Inhibition of miR-33a-3p increased Bel-7402 cell proliferation, motility, migration and invasion **A.** The expression of miR-33a-3p in Bel-7402 cells treated with miR-33a-3p inhibitor. **B–D.** The effects of inhibition of miR-33a-3p on Bel-7402 cell proliferation (B) and colony formation (C, D). **E.** The cell motility was measured using wound-healing assay. **F.** The cell migration and invasion were measured using Transwell chamber assay without (a, b) or with Matrigel (c, d). **G.** Quantitative results are illustrated for F. **H–I.** *In vivo* tumor growth was evaluated by chick embryo chorioallantoic membrane (CAM) assay (H), and tumor weights in CAM were plotted in (I). **J–K.** Tumor metastasis was identified as Dil-positive cell clones in chick embryo lung under a fluorescence microscope (J), and the quantitative results are illustrated in (K). **L.** Intravasation of Bel-7402 cells into chicken embryo lung tissues was determined by human-specific *Alu* sequence expression. *In vivo* data of represent the mean ± SD of five chick embryos.

To further understand whether miR-33a-3p is associated with cell motility and invasion of HCC cells, we inhibited the expression of miR-33a-3p using a synthesized inhibitor in low metastatic Bel-7402 cells. As shown in Figure 3A, the expression of miR-33a-3p was decreased by 58% with the miR-33a-3p inhibitor compared with the negative control (scrambled oligo). Figure 3B–3D revealed that the inhibition of miR-33a-3p in Bel-7402 cells remarkably increased cell growth (*P* < 0.001) and colony formation (*P* = 0.018).

The wound-healing assay showed that Bel-7402 cell spread was faster after miR-33a-3p inhibition (Figure 3E). As demonstrated by Boyden chamber assays (Figure 3F and 3G), cell motility and cell invasion through Matrigel were also increase significantly (*P* < 0.001).

After inhibition of miR-33a-3p expression, the growth of Bel-7402 cells on CAM was significantly promoted (inhibitor 27.0 ± 3.8 mg vs. control 14.2 ± 3.1 mg, *P* = 0.032; Figure 3H and 3I). Moreover, the metastatic cells were markedly increased after miR-33a-3p inhibition (inhibitor 7.0 ± 0.6 clones/embryo vs. control 3.3 ± 0.8 clones/embryo, Figure 3J and 3K). qRT-PCR of the human specific *Alu* sequence also demonstrated that the intravasated tumor cells into chick embryo lung tissues were significantly increased by miR-33a-3p inhibition (*P* = 0.038, Figure 3L). These data suggest that the inhibition of miR-33a-3p increases growth, motility, and invasion of HCC cells, which is in accordance with the results of miR-33a-3p over-expression.

### MiR-33a-3p directly targets 3′-UTR of *PBX3*

**Figure 4:**
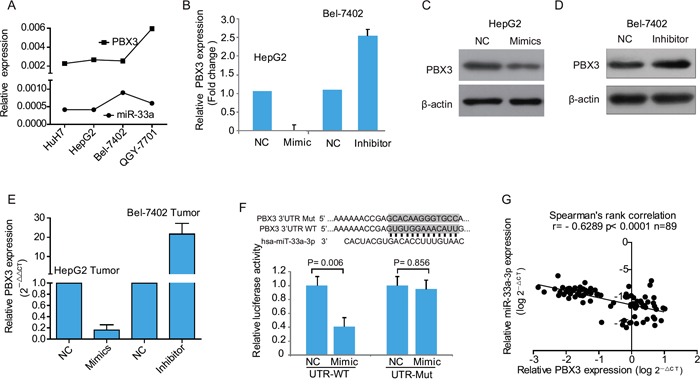
miR-33a-3p targets *PBX3* directly in hepatocellular carcinoma cells **A.** The expression levels of miR-33a-3p and *PBX3* in HCC cell lines determined by qRT-PCR. **B.** The expression of *PBX3* mRNA in HepG2 cells transfected with miR-33a-3p mimics or Bel-7402 cells transfected with miR-33a-3p inhibitor. **C–D.** The expression levels of PBX3 protein in HepG2 cells transfected with miR-33a-3p mimics (C) or Bel-7402 cells transfected with miR-33a-3p inhibitor (D). **E.** The level of *PBX3* mRNA in chick embryo chorioallantoic membrane (CAM) tumor transfected with miR-33a-3p mimics or inhibitor. **F.** Sequence alignment of human miR-33a-3p seed sequence with 3′-UTR of *PBX3* and its mutated sequence in the matched binding sites. The Firefly luciferase reporter constructs was created to detect luciferase activity in HEK-293FT cells transfected with miR-33a-3p mimics and wild-type or mutated 3′-UTR of *PBX3*. **G.** The correlation between the expression level of miR-33a-3p and *PBX3* mRNA in HCC samples (*n* = 89 cases).

To understand the mechanisms underlying the inhibitory effects of miR-33a-3p on tumor growth and metastasis in HCC cells, we performed in silico prediction of target genes of miR-33a-3p by using miRWalk 2.0. It was shown that *PBX3* gene was at the top of list with the smallest *P* value among all the predicted miR-33a-3p targets. According to the previous literatures, *PBX3* is essential for the acquisition and maintenance of the liver tumor-initiating cells properties, which is generally considered to be closely correlated with tumor migration and metastasis [17, 18]. The computational analysis revealed that miR-33a-3p bound to the 3′-UTR of *PBX3* with seed length of 12 and 13 nt. As shown in Figure 4A, the expression level of proto-oncogene *PBX3* displayed a negative correlation with miR-33a-3p in HCC cell lines. Moreover, transfection of HepG2 cells with miR-33a-3p mimics led to a significant reduction of the *PBX3* expression both at the mRNA and protein levels (Figure 4B and 4C). Up-regulated expression of *PBX3* was consistently detected after inhibition of miR-33a-3p expression in Bel-7402 cells (Figure 4D). In tumors grown on CAM, *PBX3* expression was markedly down-regulated in HepG2 cells transfected with miR-33a-3p mimics, while it was significantly increased in Bel-7402 cells transfected with the miR-33a-3p inhibitor (Figure 4E).

To determine whether *PBX3* is a direct target of miR-33a-3p, a luciferase reporter assay was performed with vectors containing 3′-UTR of *PBX3* including the putative binding sites of miR-33a-3p (Figure 4F). The corresponding mutations of binding sites were also created (Figure 4F). About 60% inhibition of luciferase activities was observed in the wild-type 3′-UTR of *PBX3* gene by mimics compared with the negative control, while this inhibition disappeared in the respective mutant constructs (Figure 4F). Hence, the miR-33a-3p binding-site in the 3′-UTR of *PBX3* is responsible for the inhibition of reporter's activity, suggesting that miR-33a-3p directly represses the expression of *PBX3* gene through its 3′-UTR.

To study the correlation between miR-33a-3p and *PBX3*, the mRNA expression levels of *PBX3* were analyzed by real-time PCR in the same set of 89 primary HCC tissues. Spearman's rank correlation analysis demonstrated that *PBX3* mRNA levels were inversely correlated with those of miR-33a-3p (*P* < 0.0001, r = −0.6289, Figure 4G), confirming that miR-33a-3p is a negative regulator of *PBX3* in HCC tissues.

### Rescue expression of *PBX3* overrides the effects of miR-33a-3p

To determine if the *PBX3* gene is required for the miR-33a-3p's effects on HCC cell invasion and metastasis, ectopic over-expression of *PBX3* was performed in HepG2 cells transfected with either the negative control or miR-33a-3p mimics.

**Figure 5:**
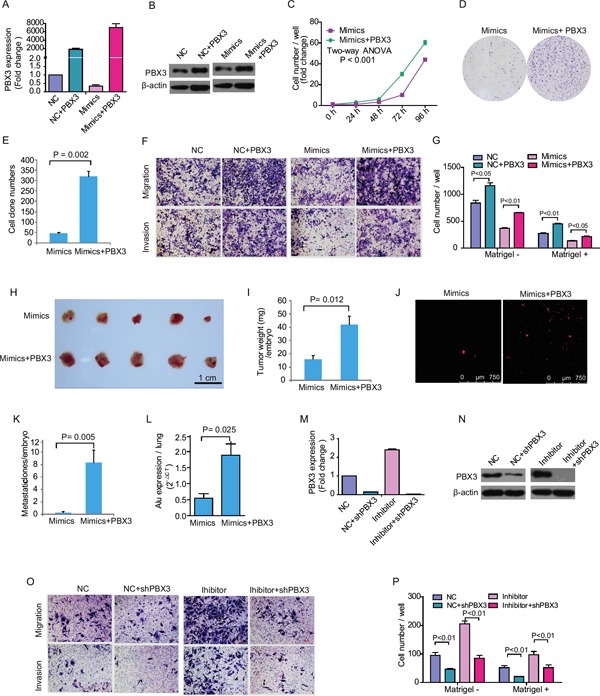
Rescue or inhibit *PBX3* expression influence growth and metastasis in hepatocellular carcinoma cell treated with miR-33a-3p mimics or inhibitor **A–B.** The expression of *PBX3* mRNA (A) and protein (B) in HepG2 cells concurrently transfected with miR-33a-3p mimics and *PBX3* lentiviruses. **C–E.** The effect of overexpression of miR-33a-3p and *PBX3* on HepG2 cells proliferation (C) and colony formation (D, E). **F–G.** The migration and invasion of HepG2 cells after concurrently transfected with miR-33a-3p mimics and *PBX3* lentiviruses (F). The quantitative results are illustrated (G). **H–I.** Over-expression of miR-33a-3p and *PBX3* in HepG2 cells on tumor growth was evaluated on chick embryo chorioallantoic membrane (CAM) assay (H). Histogram displays of tumor weights (I). **J–K.** Tumor metastasis was identified as Dil-positive cell clones in chick embryo lung under a fluorescence microscope (J), and the quantitative results are illustrated in (K). **L.** Intravasation of HepG2 cells into chicken embryo's lung tissues was determined by human-specific *Alu* sequence expression. **M–N.** The expression of *PBX3* mRNA (M) and protein (N) in Bel-7402 cells concurrently transfected with miR-33a-3p inhibitor and *shPBX3* lentiviruses. **O–P.** The migration and invasion of Bel-7402 cells after concurrently transfected with miR-33a-3p inhibitor and *shPBX3* lentiviruses (O). The quantitative results are illustrated (P).

The expression level of *PBX3* was recovered significantly after ectopic over-expression of *PBX3* in HepG2 cells, especially in those transfected with miR-33a-3p mimics (Figure 5A and 5B). Ectopic expression of *PBX3* abolished the suppressive effect of miR-33a-3p mimics on HepG2 cells growth (P < 0.001) and colony formation (*P* < 0.002) (Figure 5C-5E). Similarly, ectopic expression of *PBX3* resulted in enhanced migration and invasion of HepG2 cells transfected with either the negative control or miR-33a-3p mimics (Figure 5F and 5G). Furthermore, our *in vivo* study indicated that the inhibitory effects of miR-33a-3p on tumor growth and metastasis in the CAM model were also counteracted by transfection with *PBX3* complementary DNA (cDNA; Figures 5H-5L). These data suggest that over-expression of *PBX3* rescued the inhibitory effect of miR-33a-3p on HCC development.

Accordingly, short hairpin PBX3 (shPBX3) genes were transfected into Bel-7402 cells concurrently with the negative control or miR-33a-3p inhibitor. The *PBX3* level was considerably decreased after transfection of shPBX3 even in the presence of miR-33a-3p inhibitor (Figure 5M and 5N). It was also observed that the migration and invasion of Bel-7402 cells were further decreased after *PBX3* suppression (Figures 5O and 5P).

From positive and negative sides, the above results suggest that miR-33a-3p suppresses HCC cell growth, migration, invasion and metastasis by directly targeting *PBX3*.

## DISCUSSION

The miRNA biogenesis pathway involves the two-step process of primary miRNA transcripts (pri-miRNAs) by Drosha and Dicer enzymes, resulting in a ~22-nt miRNA/miRNA^*^ duplex product. Conventionally, the most abundant strand is defined as the mature miRNA strand (e.g., miR-33a or miR-33a-5p), whereas the less abundant strand called the miRNA star strand (e.g., miR33a^*^ or miR-33a-3p). With the in-depth research, vertebrate miRNA^*^ was demonstrated to play an important regulatory role in biological processes. In a previous study, miR-33a was shown to functions as a tumor suppressor in multiple cancers, such as melanoma, breast cancer, and osteosarcoma [19–21]. Similar to miR-33a, here, our results demonstrate that the expression levels of miR-33a-3p were not only negatively associated with the metastatic phenotype in HCC cells and tissues, but also that miR-33a-3p plays a suppressor role in cell migration and metastasis of HCC. Together, these data support a regulatory role for miR-33a-3p and suggest that miR-33 inhibits tumor cell proliferation and metastasis by both arms of the miR-33a /miR-33a-3p duplex.

Actually, the biological regulation between miRNAs and their targets is very complex *in vivo*: One miRNA may target different genes and one gene is targeted by multiple miRNAs. Research from Goedeke et al. showed that miR-33^*^ and miR-33 share the same targets involved in cholesterol efflux (ABCA1 and NPC1), fatty acid metabolism (CROT and CPT1a), and insulin signaling (IRS2) [10]. Moreover, miR-33^*^ targets the key transcriptional regulators of lipid metabolism, including SRC1, SRC3, NFYC, and RIP140 [10]. miR-33a is regarded as a tumor suppressor that targets cancer-associated genes involved in cell proliferation and cell cycle progression, such as CDK6, CCND1 [22] and Pim-1 [12]. It also inhibits tumor cell invasion or metastasis, including Twist 1 and β-catenin in non-small cell lung cancer (NSCLC) [23, 24], and HIF-1α in melanoma [19]. Whether the miR-33a-3p targets similar genes with miR-33a in growth and metastasis of HCC is still worthy of further study.

Our study identifies *PBX3* as a direct binding target of miR-33a-3p, leading to inhibition of hepatocellular tumor growth, migration and metastasis. *PBX3* is a member of the PBX family of transcription factors. Previous studies indicated a potential role of *PBX3* in carcinogenesis [25, 26]. The proto-oncogene *PBX3* is over-expressed in gastric cancer, and its expression levels are positively correlated with advanced invasion depth, clinical stage and grade of tumor differentiation [27]. Han et al. found that *PBX3* was able to reverse the suppressive effects of let-7c on colorectal cancer growth and metastasis [17]. Their group also demonstrated that *PBX3* is targeted by multiple miRNAs and is essential for tumor-initiating liver cells [18]. Accordingly, the present study demonstrates that *PBX3*, as one of the direct targets of miR-33a-3p, is able to override the suppressive effects of miR-33a-3p on HCC growth and metastasis as well. Moreover, the down-regulation of miR-33a-3p related to the increased *PBX3* mRNA levels in HCC patients at least partially predicts a higher metastasis potential and poor prognosis. Therefore, *PBX3* is functionally associated with HCC progression and can be regarded as a potential biomarker for prognosis and therapy. However, the underlying mechanisms of *PBX3* to influence cancer aggressiveness remains to be investigated. In the literature, *PBX3* is sufficient and necessary for the acquisition and maintenance of tumor-initiating cells (TIC) properties [18]. It triggers an essential transcriptional programme by activating critical genes for HCC TIC stemness that including SALL2, SOX2, CACNA2D1, WNT10A, NOTCH3, EpCAM, THY-1 and so on [18]. In the present study, we tested one of them, CACNA2D1, a previously experimentally confirmed marker of TIC as a target of PBX3, but our data failed to display reverse regulation of CACNA2D1 by miR-33a-3p. It is possible that miR-33a-3p may reduce other invasion-related transcriptional targets of *PBX3* rather than TIC-related target CACNA2D1.

Taken together, our study showed that miR-33a-3p is a potent tumor suppressor in HCC, and provided evidence that lower expression of miR-33a-3p in HCC specimens is associated with metastasis and poor survival. The suppression effect of miR-33a-3p on growth and metastasis of HCC cells is mediated, at least in part, through direct destabilization of the mRNAs of *PBX3*. Thus, these findings may help to better understand the mechanisms involved in HCC metastasis, and to discover novel and sensitive prognostic or therapeutic molecular targets for HCC.

## MATERIALS AND METHODS

### Cell lines and culture conditions

The human liver cancer cell HepG2 and human embryonic kidney HEK-293FT cell were obtained from ATCC (Manassas, Virginia, USA). HuH7, QGY-7701, and Bel-7402 cells were purchased from the Shanghai Institutes for Cell Biological Science (Shanghai, China). The cells were cultured in Roswell Park Memorial Institute (RPMI) medium 1640 (Hyclone, Logan, UT, USA) containing 10% fetal bovine serum (FBS, Gibco, Grand Island, NY, USA) and incubated at 37°C in a humidified chamber with 5% CO_2_.

### Sample of patients

Samples were collected during surgical resection at Peking University Cancer Hospital & Institute (Beijing, China) and snap frozen in liquid nitrogen. In this study, a total of 89 liver cancer and paired adjacent nontumorous tissues were included. The enrolled patients were only with one HCC tumor lesion and did not receive any preoperative chemotherapy or radiotherapy before surgery. All the cancer tissues were verified to contain at least 80% tumor cells by hematoxylin and eosin (HE) staining. Sample acquisition was approved by the ethics committee of Peking University Cancer Hospital. Informed consent documents were obtained from all of the subjects for the use of their tumors for future investigations.

### RNA extraction and quantitative RT-PCR (qRT-PCR)

Total RNA was extracted using TRIzol reagent (Invitrogen, Burlington, ON, Canada). The quantity of the RNA was determined by measuring the absorbance (Abs) at 260 and 280 nm. For mRNA detection, the cDNA was generated from 2 μg total RNA using Moloney murine leukemia virus reverse transcriptase (M-MLV RT) (Invitrogen, Carlsbad, CA). Mature miRNA was quantified using the polyA tailing method [28]. Briefly, 100 ng total RNA was added with a polyA tail by polyA polymerase (NEB, Beverly, MA, USA), followed by reverse transcription (RT) with an oligo-dT adapter primer. Data are presented as relative quantification to *U6* or *GAPDH*, based on calculations of 2^−ΔCt^ where −Δ*C*t = *C*t (Target) - *C*t (Reference). Fold change was calculated by the 2^−ΔΔCt^ method [29]. Sequences of all the primers were on the list of Supplementary Table S2.

### Transfection of miR-33a-3p mimic or inhibitor

HepG2 cells were transfected with miR-33a-3p mimic (5′-CAA UGU UUC CAC AGU GCA UCA C-3′) and Bel-7402 cells were transfected with miR-33a-3p inhibitor (5′-GUG AUG CAC UGU GGA AAC AUU G-3′) (Gene-Pharma Co. Shanghai, China) using Lipofectamine 2000 according to the manufacturer's instructions. To determine the efficiencies of miRNA mimic and inhibitor, the expression levels of miRNA in transfected cells was assessed using qRT-PCR.

### Computational prediction of miR-33a-3p target genes

Target prediction for miR-33a-3p candidates relied on complementarity between the miRNAs and putative binding sites on transcripts. Target genes of miR-33a-3p were predicted using the algorithms of the prediction database miRWalk 2.0. The prediction values were calculated to estimate the binding affinities of the miRNA and their predictive target genes. The rules for target prediction are based on the rules suggested by Allen et al. and Schwab et al. [30, 31].

### Western blotting analyses

Cells were lysed with RIPA lysis buffer and centrifuged at 12,000 rpm at 4°C. Protein concentrations were determined using the bicinchoninic acid (BCA) protein assay kit (Thermo, Rockford, USA). The samples corresponding to 20 μg of protein were resolved on a 10% denatured sodium dodecyl sulfate (SDS) polyacrylamide gel, and transferred onto a polyvinylidene difluoride (PVDF) membrane (Millipore, Bedford, MA, USA). After blocking non-specific binding sites for 1 h with 5% skim milk, the membranes were incubated with PBX3 antibodies (1: 2000 dilution; Abcam, Cambridge, UK) overnight at 4°C. Then the membranes were washed with Tris-buffered saline (TBST) and incubated with a secondary antibody for 1 h. The protein bands were detected by enhanced chemiluminescence agent (Millipore, Billerica, MA, USA). Antibody of β-Actin (1:5000 dilution; Cell Signal Technology, Beverly, MA, USA) was used as the loading control.

### Dual-luciferase reporter assay

The 3′ untranslated region (3′ UTR) of the mRNA sequence of *PBX3* containing predicted miR-33a-3p binding site was amplified by PCR. After amplification, PCR products were cloned into the pGL3.0-control (Promega, Madison, WI, USA) downstream of the luciferase coding sequence, resulting in the pGL3.0-control-3′*PBX3*. Mutation of *PBX3* was introduced in the predicted miR-33a-3p binding site by a QuikChange site-directed mutagenesis kit (Stratagene, Foster City, CA, USA). All constructs were verified by DNA sequencing.

For reporter assays, wide-type or mutant reporter constructs (300 ng) were co-transfected into HEK-293FT cells in 24-well plates with miR-33a-3p mimic or negative control (50 nM; GenePharma, Shanghai, China) and Renilla plasmid (20 ng) using Lipofectamine 2000 (Invitrogen). Firefly and Renilla luciferase activities were measured by using a dual luciferase assay (Promega, Madison, WI, USA) 24 h after transfection. Firefly luciferase values were normalized to Renilla values, and the ratio of Firefly to Renilla was presented.

### Cell proliferation, colony-forming ability, spreading, migration and invasion assays

The mock or transfected HepG2 and Bel-7402 cells were seeded into 96-well plates (1 × 10^4^ cells/well). Cell Counting Kit-8 (CCK-8; Dojindo, Tokyo, Japan) was used to assess cell proliferation according to the manufacturer's instructions. The cell clonogenic assay was performed by counting the numbers of colonies after crystal violet staining, only positive colonies (diameter > 40 μm) in the dishes were counted and compared. For the cell spreading, migration and invasion assay, 10 μg/ml mitomycin-C (Sigma, St. Louis, MO, USA) was added to exclude the effects on proliferation. Cell spreading was determined by a scratch wound healing assay as described elsewhere [32]. Briefly, a wound was incised with a pipette tip in the central area of the confluent culture on the dish. Detached cells were removed carefully with PBS and the wounded area was observed at the time of scratching and the following time point utilizing a phase-contrast Leica microscope (Leica, Wetzlar, Germany).

The cell migration or invasion assay was performed in a 24-well Boyden chamber with or without Matrigel as previously described [33]. The cells on the lower surface of the membrane were stained with crystal violet after fixation with 2% methanol for 5 min. Photographs of four randomly selected fields were taken to indicate cells that migrated to the other side of the membrane, and cell numbers were counted under a microscope.

### *In vivo* tumor growth and metastasis assay

The growth and metastatic characteristics of the cells tested *in vivo* was measured by a modified CAM assay as previously described [34]. Briefly, 5×10^6^ cells pre-stained by Dil (Molecular Probes, Eugene, OR, USA) were seeded on the CAM of each egg and incubated for 7 days. At the end of this test, the formed tumors were dissected and weighed. The lungs of chick embryos were isolated and the metastatic tumor foci (more than 20 cancer cells) were determined using a fluorescence microscope (Leica, Germany). As human genome is uniquely enriched in *Alu* sequences, the intravasated tumor cells within chicken lung tissues was detected by amplification of human-specific *Alu* sequences normalized by chicken glyceraldehyde 3-phosphate dehydrogenase (GAPDH) [35].

### Statistical analysis

All data were presented as mean ± SD and were analyzed using the IBM SPSS Statistics version 13.0 statistical package. All comparisons were analyzed with two-sided Student's t-test, unless specified. *P* < 0.05 was considered statistically significant. Experiments were repeated independently at least three times.

## SUPPLEMENTARY TABLES


