# Supplementary material for: MicroRNA-33a-3p suppresses cell migration and invasion by directly targeting PBX3 in human hepatocellular carcinoma
Source: Oncotarget. 2016 Jun 7;7(27):42461–73. doi: 10.18632/oncotarget.9886 (PMC5173148; doi:10.18632/oncotarget.9886)
Supplement: Supplementary file 1 [file oncotarget-07-42461-s001.pdf]

MicroRNA-33a-3p suppresses cell migration and invasion by directly targeting PBX3 in human hepatocellular carcinoma

SUPPLEMENTARY TABLES

Supplementary Table S1: Multivariate Cox regression analysis of clinicopathological features and miR-33a-3p expression in 85 HCC patients

Variable		Cases(n)	Multivariate COX analysis	
			RR(95%CI)	*P*-value
Sex	Male	74	0.641	0.309
	Female	11	0.273 – 1.509	
Age (year)	≤60	63	1.319	0.527
	>60	22	0.560 – 3.108	
Cirrhosis	Absent	27	0.342	0.021
	Present	58	0.138 – 0.848	
Size (cm)	≤5	45	3.014	0.008
	>5	40	1.328 - 6.843	
Venous invasion	Absent	63	0.459	0.039
	Present	22	0.219 - 0.962	
miR-33a-3p	High	46	1.108	0.776
	Low	39	0.546- 2.251	

Abbreviation: RR: Relative risk, CI: Confidence interval

Supplementary Table S2: Primers sequences

PolyA tailing qRT–PCR	
U6 forward primer	5′-CTCGCTTCGGCAGCACA-3′
U6 reverse primer	5′-AACGCTTCACGAATTTGCGT-3′
oligodT adapter primer	5′-GCGAGCACAGAATTAATACGACTCACTATAGGTTTTTTTTTTTTVN-3′
Reverse primer	5′-GCGAGCACAGAATTAATACGAC-3′
miR-33a-3p forward primer	5′-5′- CAATGTTTCCACAGTGCATCAC-3′
Q-PCR analysis of PBX3 and Alu	
PBX3 forward primer	5′-CAAGTCGGAGCCAATGTG-3′
PBX3 reverse primer	5′-ATGTAGCTCAGGGAAAAGTG-3′
Human GAPDH forward primer	5′-GACCCCTTCATTGACCTCAAC-3′
Human GAPDH reverse primer	5′-CTTCTCCATGGTGGTGAAGA-3′
Alu forward primer	5′ACGCCTGTAATCCCAGCACTT-3′
Alu reverse primer	5′TCGCCCAGGCTGGAGTGCA-3′
Chicken GAPDH forward primer	5′ACGCCATCACTATCTTCCAGGAG-3′
Chicken GAPDH reverse primer	5′TACTTACCCCAGCCTTCTTG-3′
PGL-control 3′-UTR recombinant plasmid construct	
PBX3 3′-UTR forward primer	5′-TGCTCTAGACCGGTCTCTGGCCACACTTTTCCCTG
PBX3 3′-UTR reverse primer	5′-CGGAATTCATGCATACATATAACTACCACGAGGAAGAC
Mut–PBX3 forward primer	5′- CGAGCACAAGGGTGCCA GGTCTTAATTCAACACAGGATCG
Mut–PBX3 reverse primer	5′- GACCTGGCACCCTTGTGCTCGGTTTTTAAAAGTTGCAAG
